# Knowledge of and willingness to take pre-exposure prophylaxis among men who have sex with men in Israel

**DOI:** 10.1186/s13584-021-00500-x

**Published:** 2021-12-06

**Authors:** Mor Zohar, Shilo Guy, Levy Itzchak

**Affiliations:** 1 Tel Aviv Department of Health, Ministry of Health, 12 Ha’arba’a Street, Tel Aviv, Israel; 2 School of Health Sciences, Ashkelon Academic College, Ashkelon, Israel; 3 School of Social Work, Faculty of Social science, Tel Aviv University, Tel Aviv, Israel; 4 AIDS and STD Unit, Sheba Medical Center, Tel Hashomer, Israel; 5 Faculty of Medicine, Tel Aviv University, Tel Aviv, Israel

**Keywords:** Gay men, HIV, Post-exposure prophylaxis, Sexual behavior

## Abstract

**Background:**

Oral pre-exposure prophylaxis (PrEP) with tenofovir disoproxil fumarate and emtricitabine (TDF/FTC) has been found to reduce viral acquisition among HIV-negative MSM. This cross-sectional study was conducted before pre-exposure prophylaxis (PrEP) licensure in Israel, and aimed to compare men who have sex with men (MSM) who had heard of PrEP with those who had not, as well as MSM willing to take PrEP with those who were hesitant or not willing to take PrEP.

**Methods:**

HIV-negative MSM responded anonymously to questionnaires in 2017 regarding their knowledge of and willingness to take PrEP, prior use of PrEP and post-exposure prophylaxis (PEP), and their sexual behaviors.

**Results:**

Among 1705 participants, 1431 (83.9%) had heard about PrEP. They were older and more often reported being Jewish, having an academic degree, self-identifying as gay/bisexual, being tested for HIV in the last year, participating in group sex, using alcohol or drugs before or during sex, and having prior use of PrEP/PEP compared with MSM who had not heard about PrEP. A total of 760 (44.8%) participants indicated that they would consider taking PrEP, 567 (33.5%) maybe would consider taking PrEP, and 367 (21.7%) would not take PrEP. Those who were willing to take PrEP had a lower level of education, were involved in high-risk sexual behaviors, used alcohol or drugs before or during sex, and had previously used PrEP/PEP compared with participants who maybe would consider taking or would not take PrEP. When participants were asked to indicate if they were willing to take PrEP at different potential efficacies and costs, the willingness to using PrEP increased with the potential efficacy of the drug and adversely related to its cost.

**Conclusions:**

PrEP awareness was high, and 44.8% indicated willingness to take PrEP, especially those who reported high-risk sexual behaviors. This supports the current policy in Israel to allow PrEP to MSM who are at high-risk. In order to maintain a high level of PrEP-adherence, physicians should consider structural barriers, such as negative stigma of being promiscuous, lack of perceived HIV-risk, difficulties in accessing clinics or paying for PrEP, inability to follow-up or low tolerability of the medication.

**Supplementary Information:**

The online version contains supplementary material available at 10.1186/s13584-021-00500-x.

## Background

Men who have sex with men (MSM) are affected disproportionately by HIV in Israel and other high-income countries [1]. A wide array of efforts, including behavioral interventions and encouraging frequent HIV testing and the use of barrier methods such as condoms, have been employed among MSM to decrease their exposure to sexual risks [2]. Following the introduction of antiretroviral treatment (ART), scientific evidence has demonstrated the benefits of treatment as prevention and that the reduction of patients' HIV viral load decreases the risk of viral transmission in susceptible social/sexual networks [3]. Recently, the use of oral pre-exposure prophylaxis (PrEP) with combined tenofovir disoproxil fumarate 300 mg and emtricitabine 200 mg (TDF/FTC) has been found to reduce viral acquisition among HIV-negative MSM [4].

This biomedical breakthrough of using PrEP to reduce significantly the risk of acquiring HIV was confirmed by the US Food and Drug Administration’s approval of TDF/FTC for PrEP in HIV-negative adults at high risk [5] and the release of new guidelines by the World Health Organization [6] and the U.S. Centers for Disease Control and Prevention [7]. Daily use of PrEP was approved in Israel in September 2017 for a subset of MSM who engage in high-risk sexual behavior [8], while efforts to encourage this population to use condoms have continued.

The success of PrEP depends on the availability of the drug, convenience of use, and its acceptability by the community. A recent meta-analysis reported an estimate of PrEP acceptability of 57.8% among MSM [9]. In Israel, data are lacking regarding MSM’s knowledge of PrEP and their willingness to use PrEP, although the rate of HIV acquisition among MSM is disproportionately high. Among all males diagnosed with HIV in Israel between 1981 and 2017, the proportion of MSM was 38.6% [10].

The purpose of this study was to compare the number of MSM who had heard about PrEP with those who had not heard about PrEP. In addition, we aimed to compare MSM who were willing to take PrEP with those who maybe would take or were not willing to take PrEP. This study was performed prior to the licensure of PrEP in Israel to allow policy stakeholders and community leaders to estimate the potential number of MSM who are interested in taking PrEP, and assess their behavioral characteristics, as an important step toward increasing PrEP utilization.

## Methods

### Participants and procedures

This cross-sectional study included HIV-negative MSM recruited between May and July 2017 through geosocial-networking smartphone applications (Grindr, Atraf), which are commonly used by MSM to meet potential sexual partners, as well as through virtual social networks (Facebook). Application users received pop-up advertisements with a link to the survey. After signing informed consent forms, participants were asked to reply anonymously to a set of 52 questions regarding their demographic characteristics, whether or not they had heard about PrEP, prior PrEP or post-exposure prophylaxis (PEP) use, and their sexual behaviors during the last year (Additnal file 1: Appendix). In order to encourage participants to respond to the questionnaire, a raffle was performed, awarding the winners with annual gym subscriptions.

### Measures

High-risk sexual behavior was measured by the HIV Incidence Risk Index for MSM (HIRI-MSM) score [11], which includes age and the following criteria concerning the last six months: the total number of male sex partners and HIV-positive partners, the number of condomless receptive anal sex encounters with any HIV status partner, the number of encounters that included insertive anal sex with an HIV-positive partner, and also the use of poppers or amphetamines. Participants were scored between 0 and 47, and a score of 10 or greater suggests high sexual risk.

### Variables

Dependent variables for this study were PrEP awareness, which was assessed by asking participants if they had heard about PrEP (yes/no), and willingness to take PrEP, which was assessed by asking participants whether they would consider taking PrEP (yes/maybe/no). Independent variables include demographic characteristics, sexual behavior during the last year, previous use of PEP or PrEP, and their attitudes towards potential sexual risks.

### Statistical analysis

Independent variables were compared using Chi-square or Student's *t*-tests for categorical and continuous variables, respectively. Variables whose *p* values were less than 5% in the univariate analyses were included in the multivariate analysis to identify attributes predicting willingness to take PrEP.

## Results

Between May and July 2017, 2645 responders started answering the questionnaire: 2472 (93.5%) were recruited through smartphone applications and 173 (6.5%) through Facebook. Of these, 914 did not complete the questionnaire, 12 did not have sex in the last year, nine had sex only with women, and five provided inconsistent responses. The average age of the 1705 MSM who met the inclusion criteria was 32 years (range 18–77). Among all participants, 1561 (91.5%) were born in Israel, 1594 were Jews (93.5%), 957 (56.1%) had an academic degree, 1423 (83.5%) self-identified as gay or bisexual, and 409 (23.9%) had a steady partner.

Overall, 1431 (83.9%) had previously heard about PrEP (Table 1). Those who had heard about PrEP were older and more often reported being Jewish, having an academic degree, and self-identifying as gay or bisexual compared with MSM who had not heard about PrEP. Those who had heard about PrEP more often reported having been tested for HIV in the last year, participating in group sex, and using alcohol or drugs before or during sex, and less often reported having concurrent sex with women. In addition, those who had heard about PrEP more often reported previous use of PrEP or PEP and that they would have condomless anal intercourse with an HIV-positive partner who is taking ART.

**Table 1:** Comparison of characteristics of MSM who had heard about or had not heard about pre-exposure prophylaxis

Characteristics	Heard of PrEP N = 1431 (%)	Had not heard of PrEP N = 274 (%)	*P*
*Demographics*			
Age in years ± SD	33.7 ± 8.7	32.1 ± 10.8	0.02
Israeli born	1315 (91.8)	246 (89.7)	0.2
Jewish	1350 (95.1)	244 (90.4)	0.003
Academic degree	842 (58.9)	115 (42.0)	< 0.001
Self-definition as a gay or bisexual ma	1232 (86.2)	191 (71.5)	< 0.001
*Sexual behaviors in the past year*			
HIV test in the past year	1358 (95.0)	242 (89.3)	< 0.001
Sex with both men and women	127 (10.2)	54 (19.9)	< 0.001
Age at first anal sex	18.3 ± 4.6	18.3 ± 5.1	0.9
Current steady partner	347 (24.3)	62 (23.3)	0.7
Open steady relationships	263 (73.8)	31 (50.0)	< 0.001
CAI with partner whose HIV status was unknown/discordant	573 (40.6)	111 (41.7)	0.7
Prefers receptive anal sex	812 (57.2)	145 (56.0)	0.7
Participation in group sex	563 (40.5)	88 (33.5)	0.04
Special sexual repertoire^a^	355 (27.5)	58 (26.4)	0.6
Sexual risk behavior score^b^	17.0 ± 9.5	16.6 ± 9.5	0.5
Previous STD diagnosis	279 (21.5)	44 (18.9)	0.4
Alcohol or drug use before or during sex	901 (69.4)	130 (56.3)	< 0.001
*PrEP/PEP use*			
Previous use of PEP	84 (5.8)	1 (0.4)	< 0.001
Previous use of PrEP	236 (16.5)	19 (6.9)	< 0.001
*Attitudes*			
My sexual behavior is riskier than that of my friends	223 (15.6)	50 (19.0)	0.2
I am generally more horny than my friends	515 (36.4)	97 (36.3)	0.9
I can have CAI with an HIV-positive partner who takes antiretroviral treatment	453 (32.3)	45 (17.4)	< 0.001

*CAI* condomless anal intercourse, *PEP* post-exposure prophylaxis, *PrEP* pre-exposure prophylaxis, *SD* standard deviation, *STD* sexually transmitted disease

^a^Urination, fisting, sado, bondage

^b^HIV Incidence Risk Index for MSM (HIRI-MSM) score

Of the 1694 participants who responded regarding their willingness to take PrEP, 760 (44.8%) indicated that they would consider taking PrEP, 567 (33.5%) maybe would consider taking PrEP, and 367 (21.7%) would not consider taking PrEP (Table 2). Those who were willing to take PrEP were less likely to have an academic degree, but more likely to have been tested for HIV in the past year, report earlier anal sex, have condomless anal intercourse with a partner whose HIV status was unknown or discordant, to prefer receptive anal intercourse, participate in group sex and other sexual repertoire, have a higher HIRI-MSM risk score, use alcohol or drugs before or during sex, and to have previously used PrEP or PEP compared with participants who maybe would take PrEP and those who would not consider taking it. MSM who were willing to take PrEP reported taking more sexual risks and having a greater sexual appetite than their friends. They also reported that they would have condomless anal intercourse with an HIV-positive partner who is taking ART. MSM who were willing to take PrEP were less likely to be diagnosed with an STD in the past year than those who did not consider taking PrEP. In the multivariate analysis, a lower level of education, having condomless anal intercourse with a partner whose HIV status was unknown or discordant, preferring receptive anal sex, participating in group sex and other sexual repertoire, having a higher HIRI-MSM risk score, using alcohol or drugs before or during sex, previously using PrEP or PEP, and taking more sexual risks were associated with willingness to take PrEP.

**Table 2:** Comparison of characteristics of MSM who would consider taking PrEP with those who maybe or would not consider taking PrEP

Univariate					Multivariate^	
Characteristics	Would consider taking PrEP N = 760 (44.8%)	Maybe would consider taking PrEP N = 567 (33.5%)	Would not consider taking PrEP N = 367 (21.7%)	*P*	OR (95% CI)	*P*
*Demographics*						
Age, years ± SD	33.8 ± 9.4	32.7 ± 8.7	33.6 ± 8.8	0.09		
Israeli born	689 (90.0)	520 (91.7)	341 (93.4)	0.4		
Jewish	713 (94.4)	525 (93.4)	346 (94.6)	0.5		
Academic degree	361 (47.5)	322 (56.9)	230 (63.0)	0.005	0.8 (0.6–0.9)	0.02
Self-definition as a gay or bisexual man	638 (84.1)	466 (82.2)	309 (84.6)	0.5		
*Sexual behaviors in the past year*						
HIV test in the past year	729 (96.0)	530 (93.6)	331 (90.5)	0.001	1.5 (0.9–2.3)	0.1
Sex with both men and women	79 (10.5)	61 (10.8)	40 (10.9)	0.9		
Age at first anal sex	17.8 ± 4.9	18.3 ± 4.3	19.1 ± 4.8	< 0.001	1.0 (0.9–1.1)	0.6
Current steady partner	182 (24.0)	131 (23.4)	93 (25.8)	0.7		
Open steady relationships	142 (78.1)	85 (64.9)	62 (66.7)	0.02		
CAI with partner whose HIV status was unknown or discordant	403 (54.6)	206 (36.8)	72 (20.3)	< 0.001	3.7 (2.5–5.6)	0.01
Prefers receptive anal sex	458 (60.9)	183 (32.3)	202 (56.4)	< 0.001	1.4 (1.1–1.7)	0.04
Participation in group sex	357 (51.5)	107 (21.1)	115 (34.5)	< 0.001	1.4 (1.1–1.8)	0.01
Special sexual repertoire^a^	225 (34.0)	65 (12.9)	77 (26.4)	< 0.001	1.3 (1.2–1.4)	0.04
Sexual risk behavior score^b^	19.9 ± 10.1	16 ± 8.5	12.6 ± 7.5	< 0.001	1.2 (1.1–1.4)	0.04
Previous STD diagnosis	167 (24.5)	51 (10.2)	105 (31.8)	< 0.001	1.0 (0.8–1.3)	0.9
Alcohol or drug use before or during sex	519 (75.0)	327 (65.6)	185 (57.0)	0.005	1.4 (1.2–1.8)	0.002
*PEP/PrEP use*						
Previous use of PrEP	156 (20.5)	35 (6.1)	64 (17.3)	< 0.001	1.5 (1.1–2.0)	0.02
Previous use of PEP Did not use PEP	81 (10.7)	4 (0.7)	0 (0)	< 0.001	13.9 (5.0–39.2)	< 0.001
*Attitudes*						
My sexual behavior is riskier than that of my friends	168 (22.3)	67 (12.0)	38 (11.2)	< 0.001	1.7 (1.3–2.3)	< 0.001
I am generally more horny than my friends	296 (40.0)	208 (37.6)	108 (30.4)	0.008	1.1 (1.0–2.3)	0.4
I can have CAI with an HIV-positive partner who takes antiretroviral treatment	452 (61.3)	29 (5.3)	16 (4.5)	< 0.001		

*CAI* condomless anal intercourse, *PEP* post-exposure prophylaxis, *PrEP* pre-exposure prophylaxis, *SD* standard deviation, *STD* sexually transmitted disease

^^^Would versus maybe/would not consider taking PrEP

^a^Urination, fisting, sado, bondage

^b^HIV Incidence Risk Index for MSM (HIRI-MSM) score

Of all 1431 who had heard about PrEP, the majority (N = 779, 54.4%) had learned about PrEP from the Internet and only a few (N = 24, 1.7%) through their physician. The majority of participants (N = 1046, 73.0%) preferred the PrEP regimen to be provided by their family physician, followed by the AIDS Task Force (the most significant AIDS-related non-governmental organization in Israel, N = 520, 36.4%) or by AIDS treatment centers in hospitals (N = 307, 21.5%).

Previous use of PrEP was reported by 255 (14.9%) participants. Of these, 129 (50.6%) bought it online, 69 (27.1%) were prescribed PEP but ultimately used it as PrEP, and 57 (22.4%) received the drug from an HIV-positive friend. Previous use of PEP was reported by 85 (5.0%) participants. Of these, 69 (81.2%) were prescribed PEP more than once and 46 (66.7%) were advised to take PEP and started the therapy, but did not complete the monthly course.

The willingness of study participants to use PrEP increased with the potential efficacy of the drug to prevent HIV infection, as it was presented in the questionnaire, and adversely related to its various costs (Table 3). More than half were willing to use PrEP if the efficacy had been > 95% at a monthly cost of 500 NIS (~ 120 Euro).

**Table 3:** Willingness to take PrEP among those who have heard about the prevention, by success of the technology and its price

	Want PrEP	Do not want PrEP	*P*
If PrEP is 80% successful	401 (60.2)	174 (23.1)	< 0.001
If PrEP is 90% successful	575 (86.1)	270 (35.7)	< 0.001
If PrEP is 95% successful and costs 1000 NIS	183 (27.5)	79 (10.5)	< 0.001
If PrEP is 95% successful and costs 500 NIS	356 (53.7)	163 (21.7)	< 0.01
If PrEP requires monthly visits + blood tests	449 (67.9)	162 (21.6)	< 0.001

*IS* New Israeli Shekel (1 NIS ~ 0.25 Euro); *PrEP* pre exposure prophylaxis

## Discussion

Among all study participants, 83.9% had heard about PrEP. Of all the 1694 participants who responded regarding their willingness to take PrEP, 44.8% reported that they were willing to take PrEP, and 33.5% indicated that they maybe would consider taking it. These figures are comparable with studies conducted in other high-income countries, showing 48% willingness in the United Kingdom [12], 55% in Canada [13], 65% in Germany [14]-depending on the drug's availability, cost, and the time and methods of the study.

In our study, participants who were willing to take PrEP were involved in higher-risk sexual behaviors compared with those who maybe would consider taking PrEP and those who would not consider taking PrEP, as reported elsewhere [15]. As 40.1% of all study participants reported that they had engaged in condomless anal sex with a partner whose HIV status was unknown or discordant in the last year, PrEP is a valuable risk management strategy among those men. Although PrEP has successfully prevented HIV transmission, its availability may lead to increased risk behaviors, which may result in possible exposure to STDs [16]. However, MSM who access PrEP are under continuous medical supervision. Providers who prescribe PrEP have the opportunity to weigh the sexual health of MSM against their pleasure-seeking behavior by supporting PrEP adherence, maintaining continued dialogue related to behavioral risk reduction, and recommending routine counseling and testing for STDs, thus limiting further transmission [17].

Although PrEP was not available in Israel when this study was conducted, ~ 15% of the participants had used off-label PrEP at least once and approximately half of those purchased the drug online, making it likely that they have not received appropriate and regular medical check-ups. Although this high level of awareness could be viewed as a proxy for higher sexual health literacy or greater access to sexual health information, these drugs have not been supplied by a registered pharmacy and are not regulated. Thus, their pharmacological qualities might differ from those of the licensed drug and, consequently, may not provide complete protection.

Previous use of PEP was reported by ~ 5% of the participants in this study. Many of these users sought PEP from a medical care clinic, asking for it due to an alleged incident of potential exposure, but actually intending to use it as PrEP. The availability of PrEP in Israel will probably make this practice unnecessary. This use of PrEP before its licensure in Israel was high compared to the results of the European Men-Who-Have-Sex-With-Men Internet Survey (EMIS-2017) study performed in 2017 Europe and Israel, and the average use was 3.0% among the total sample of ~ 173,000 MSM [18].

More than 80% of the participants in this study had heard about PrEP and ~ 45% reported that they were willing to take the drug. The high number of men who would consider taking PrEP may be informative to medical insurers, healthcare providers, and drug suppliers in evaluating future interest and estimating PrEP uptake. In order to assess the potential number of PrEP users, we used an estimation of 94,176 (95% CI 92,562–96,063) self-identified Jewish gay/bisexual men aged 18–44 years living in Israel [19]. Previous research has found that high-risk MSM were twice as likely to respond to convenience surveys and report more risk behaviors compared with probability-based surveys [20]. In order to adjust for over-reporting, we applied a 50% correction factor to the proportion of participants in this study who reported that they would consider taking PrEP (22.4%), thus we estimated that 21,095 (95% CI 20,733–21,518) MSM will potentially be interested in using PrEP in Israel.

Most participants indicated that they would prefer to get the PrEP prescription from their primary care provider rather than from AIDS treatment centers or AIDS organizations. In order to implement this PrEP service, dedicated physicians and clinic staff should be trained to respond in a culturally competent and non-stigmatizing way to the upcoming influx of MSM inquiring about PrEP. Providers should be knowledgeable about common high-risk behaviors in the MSM community, possible side effects of the drug, and its proper use.

Men in our study were asked to indicate their willingness to take PrEP in different potential efficacies as were presented in the questionnaire and at various costs. As expected, higher efficacy and lower cost increase the willingness. Interestingly, participants were more sensitive to the price of the drug rather than its potential efficacy, and halving the price doubled the willingness. In order to reduce the financial constraints and decrease the incentive of MSM to purchase PrEP online, an alternative route should be generated to provide the drug to men who are unable to bear the copayment. Since our study was performed, new information shows the effectiveness of PrEP is higher than 95%, as it was originally presented to the participants during the study and the price has been reduced. Therefore, the willingness to take PrEP may be underestimated in our study. As we learned from the recent COVID-19 outbreak [21], the use of PrEP may also be reduced during a period characterized by a social atmosphere of insecurity and apprehension.

Public health interventions aiming to promote PrEP among MSM should consider the knowledge gap found among MSM in this study. This study showed that MSM who had not heard of PrEP were younger, non-Jewish, and had a lower level of education. These MSM may also be less exposed to other safe-sex messages and, therefore, can be reached through smartphone applications, which are cheap, widespread, and allow users to maintain anonymity.

Oral PrEP is an effective biomedical method used to prevent HIV infection, adding to condoms and other harm reduction strategies. As the success of the treatment depends on appropriate use and adherence, health providers should be attentive to possible structural barriers that exist for MSM to access the treatment, such as negative stigma of being promiscuous, lack of perceived HIV risk, difficulties in accessing clinics or paying for the drug, inability to manage the multiple follow-up visits or low tolerability of the medication [22]. Several strategies have been proposed to achieve a large scale of PrEP coverage among MSM. First, more family doctors should advise PrEP to MSM who are at high sexual risk. Doctors should be informed of the updated follow-up recommendations and the possible side effects of the medication. In addition, PrEP should be integrated with STD screening clinics and HIV testing sites. Second, an alternative "on demand" course can be proposed to MSM who are discouraged by the daily pill burden. The alternative drug regimen has at least comparable effectiveness to daily use [23]. Third, PrEP education material should be distributed by community-based organizations and health insurers to empower MSM to take control over their health. These interventions should address possible stigma related to PrEP use. This may be especially valid among marginalized or sub-urban MSM populations or minorities, such as Arab [24]. Fourth, financial barriers should be eliminated. The Israeli AIDS task force and a pharmaceutical company provide free PrEP to MSM who face financial difficulties. Last, medical insurers should deploy policy-level solutions to promote sexual health in community clinics and HIV prevention interventions. This includes training programs for physicians, encouraging them to raise the issue of sexual orientation while taking medical history and discuss possible consequences of high-risk sexual risk behavior with their patients. In order to promote HIV-prevention, medical insurers should also encourage family physicians to propose PrEP proactively and reassure that those who are using PrEP are being followed-up.

### Limitations

This is the first study conducted in Israel that assesses knowledge of and willingness to use PrEP, yet it is subject to several limitations. First, this study included a convenience sample in Israel, making the representativeness of MSM who participated in the study difficult to assess and limiting its generalizability to other countries. Of note, the population which responded appeared to be engaged in high-risk sexual behavior, thus making our assumptions appropriate for high-risk MSM. Second, all collected data were self-reported by participants and may be subject to bias, including social desirability and recall bias. In order to minimize these biases, a computer-administered questionnaire was used, reducing pressure for favorable reporting while ensuring that most questions in the study pertained to the last 6–12 months.

## Conclusions

PrEP awareness was high in this study and 44.8% of participants indicated that they were willing to take PrEP, especially MSM who were involved in high-risk sexual behaviors. The social acceptability of PrEP in Israel is likely to have increased since its approval in September 2017. The findings of this study support Israel's current policy to allow MSM who are at high sexual risk to take PrEP under medical follow-up. However, further studies should evaluate the awareness of and willingness to use PrEP over time and assess the actual uptake of PrEP, its positive consequences, and possible adverse outcomes (e.g., greater exposure to STDs).

## Supplementary Information

**Additional file 1**. Study Questionnaire.

## Data Availability

The datasets used and/or analyzed during the current study are available from the corresponding author on reasonable request.
