# Supplementary material for: Knowledge of and willingness to take pre-exposure prophylaxis among men who have sex with men in Israel
Source: Isr J Health Policy Res. 2021 Dec 6;10:71. doi: 10.1186/s13584-021-00500-x (PMC8647505; doi:10.1186/s13584-021-00500-x)
Supplement: Supplementary file 1 — Additional file 1. Study Questionnaire. [file 13584_2021_500_MOESM1_ESM.docx]

**Supplementary Appendix - Survey questionnaire**

1. What year were you born?
2. Born in Israel? (yes/no)
3. What country were you born in?
4. In what year did you immigrate to Israel?
5. What is your religion? (Jewish/Muslim/Christian/Druze/Other____)
6. What is your average monthly income?
7. What is your higher education degree? (High-school/high-education with no academic degree/academic first degree/academic second degree or higher)
8. How do you define yourself in terms of sexual orientation? (gay/bisexual/straight)
9. When was your last HIV test? (past 6 months/past year/past 2 years/more than two years ago/never)
10. What was the result of your last HIV test? (negative/positive)
11. What was your age of first sexual intercourse (specify the age of first oral sex and the age of first anal sex) with a man?
12. With whom did you have sexual intercourse in the past year? (men only/ women only/ both men and women/I did not have sexual intercourse in the past year)
13. Do you have a steady partner in the past year? (yes/no)
14. (If yes on Q13): Have you had a concomitant non-steady partner in the last year?
15. In the past year, did you engage in anal intercourse with a guy (top or bottom) without using a condom? (yes/no)
16. How many times did you engage in anal intercourse with a guy without using a condom in the past year? (once/2-5 times/6-10 times/11 times or more)
17. On these occasions, where you engaged in anal intercourse with a guy without using a condom, what were your sexual preferences? (I was topping/I was bottoming/I was versatile: both topping and bottoming)
18. What is your usual sexual preference in the past year? (I am usually the bottom/I am usually the top/I am usually versatile/I usually engage in oral rather than anal sex)
19. In the past year, did you engage in anal intercourse with a man that you knew to be HIV positive? (yes/no/I do not know)
20. On these occasions, where you had anal intercourse with a man that you knew to be HIV positive, what did the sexual activity include? (I was topping/I was bottoming/I was versatile: both topping and bottoming)
21. In the past year, did you engage in condomless anal intercourse with a man whose HIV status you did not know, or whose HIV status you knew was discordant with yours? (yes/no/I do not know)?

How many time? (once/2-5 times/6-10 times/11 times or more)

1. Does the issue of HIV come up with your sexual partners prior to intercourse? (most of the time/some of the time/rarely)
2. Who usually raises the issue of HIV? (usually me/usually my sexual partner/sometimes me and sometimes my sexual partner)
3. Were you engaged in a group sex in the past year? (yes/no)
4. Were you engaged in a sexual activity which included special repertoire in the past year (such as: fisting, urination [water-sports], BDSM [bondage, domination, sadomasochism])? (yes/no)
5. Did you ever take pre exposure HIV prophylaxis (PrEP)? (yes/no)
6. How did you get the treatment? (from an HIV-positive friend/I bought it abroad/I turned to a clinic to get PEP and used it as PrEP/ other____)
7. Did you ever take post exposure HIV prophylaxis (PEP)? (yes/no)
8. Did you fulfill the PEP treatment (full month)? (yes/no)
9. (if did not fulfill treatment) why did you not fulfill the PEP treatment? (because of the side effects/ because I was not in high risk/ because I forgot)
10. Did you ever hear of pre exposure HIV prophylaxis (PrEP)? (yes/no)
11. How did you hear of PrEP? (through my friends/through the internet or media/through my doctor)
12. Would you consider taking PrEP? (yes/maybe/no)
13. If PrEP treatment would protect you in only 90%, would you consider taking it? (yes/no)
14. If PrEP treatment would protect you in only 80%, would you consider taking it? (yes/no)
15. If PrEP treatment would protect you in 95%, but will cost 1000 New Israeli Shekel a month, would you consider taking it? (yes/no)
16. If PrEP treatment would protect you in 95%, but will cost 500 New Israeli Shekel a month, would you consider taking it? (yes/no)
17. If PrEP treatment would protect you in 95%, will you continue using a condom? (always/usually yes/usually no/no)
18. If PrEP treatment will require a monthly check-up by a doctor, including blood tests and disclosing your sexual behavior, would you consider taking it? (yes/maybe/no)
19. What would you prefer: Pre exposure treatment (PrEP; taken daily), or Post exposure treatment (PEP; taken one month after supposed exposure to HIV). Do note that PEP is less efficient than PrEP. (PrEP/PEP)
20. Who do you prefer to supply PrEP? (HIV centers in hospitals/family physician/HIV task-force/private doctors).
21. What is embarrassing about PEP? (multiple choices: asking for it from a physician is embarrassing/ fear of medical tolerance (and then, if I will be infected, treatments will be less effective)/ drug cost (it will not be for free)/ the need for monthly check-up, including blood tests and disclosing my sexual behavior to the provider/ side effects/ embarrassment/ other ____)
22. If you take PrEP, will you notify your sexual partners? (yes/no)
23. Were you diagnosed with an STD in the past year? (yes/no)
24. (if yes on Q44) with which STD were you diagnosed? (syphilis/gonorrhea/condyloma acuminata/I do not know the name)
25. Are you hornier compared to your friends? (hornier/like my friends/less horny)
26. Compared to your friends, do you take more sexual risks? (take more sexual risks/like my friends/take less sexual risks)
27. Have you used alcohol or drugs prior to or during sexual intercourse in the last year? (always/often/rarely/never)
28. Please list all the substances you use (you can list more than one): (alcohol to intoxication levels/poppers/MDMA/speed/heroin/GHB/Ketamine)
29. Would you have condomless anal sex with an HIV-positive person who is under antiretroviral medications? (medication given to HIV-positive persons)? (yes/it depends/no)
30. When you are abroad, do you take more sexual risks than in Israel? (yes, I take more sexual risks abroad/the same/I take less sexual risks while abroad)
31. Do you think that porn movies when actors engage in bareback practices (anal intercourse without a condom) impact your sexual behavior? (these movies encourage me to have bareback practices/these porn movies do not change my sexual practices/contrary: these movies encourage me to use condoms/other ____)

MDMA, 3,4-Methyl​enedioxy​methamphetamine; GHB, gamma-Hydroxybutyric acid.
